# French patients on daily hemodialysis: clinical characteristics and treatment trajectories

**DOI:** 10.1186/s12882-016-0306-7

**Published:** 2016-07-29

**Authors:** Adélaïde Pladys, Sahar Bayat, Anne Kolko, Clémence Béchade, Cécile Couchoud, Cécile Vigneau, on behalf of the REIN registry

**Affiliations:** 1 EHESP Rennes, Sorbonne Paris Cité, Rennes, France; 2 Université Rennes 1, UMR CNRS 6290, Rennes, France; 3 EHESP Rennes, Sorbonne Paris Cité, EA MOS, Rennes, France; 4 Association AURA, Paris, France; 5 CHU Caen, Service de néphrologie, Caen, France; 6 Université de Caen Normandie, 1086 INSERM, Caen, France; 7 Registre REIN, Agence de la biomédecine, Saint Denis La Plaine, France; 8 CHU Pontchaillou, Service de néphrologie, Rennes, France

**Keywords:** Daily hemodialysis, End stage renal disease, Patient profiles, Patients trajectories, REIN registry

## Abstract

**Background:**

Increasing the weekly frequency of hemodialysis sessions has positive effects, on the control of several biological data of patients. However, knowledge about Daily HemoDialysis (DHD) practices is limited in France. The aim of the present study was to describe the characteristics and treatment trajectories of all French patients undergoing DHD.

**Methods:**

All patients older than 18 years who started DHD between 2003 and 2012 in France were included and followed until December 31, 2013. The patients’ demographic and clinical characteristics and treatment modalities were extracted from the French Renal Epidemiological and Information Network (REIN) registry.

**Results:**

During the inclusion period, 753 patients started DHD in France. Based on their median age (64 years), patients were classified in two groups: “old” group (≥64 years) and “young” group (<64 years). Patients in the old group had more comorbidities than in the young group: 48 % had diabetes (*vs* 29 % in the young group), 17 % an active malignancy (*vs* 10 %) and 80 % ≥1 cardiovascular disease (vs 41 %). Concerning patients’ treatment trajectories, 496 (66 %) patients started with another dialysis before switching to DHD and 257 (34 %) directly with DHD. At the end of the follow-up, 69 % of patients in the old group were dead (27.4 % in the young group) and kidney transplantation was more frequent in the young group (30.4 % vs 0.5 %).

**Conclusion:**

In France, DHD is proposed not only to young in rather good clinical conditions and waiting for kidney transplantation, but also to old and frail patients with higher mortality.

**Electronic supplementary material:**

The online version of this article (doi:10.1186/s12882-016-0306-7) contains supplementary material, which is available to authorized users.

## Background

End Stage Renal Disease (ESRD) is a chronic progressive disease and a major public health issue as indicated by the dramatic increase in the number of patients treated for ESRD worldwide and also in France [1, 2].

Hemodialysis (HD), three times per week, is the most frequent renal replacement therapy (RRT) [3]. However, in recent years, new HD regimens characterized by changes in the weekly frequency [4–9], the session’s duration [6–10] or the dialysis doses [8, 10–14] have been assessed. The main aim of these modifications was to reduce the side effects of thrice-weekly HD [4, 8, 15] that are caused by the fluctuations in the level of urea particles and their toxicity between HD sessions [4, 6, 15–19], and also to improve the patients’ quality of life [4, 8, 10, 11, 20, 21] by decreasing the number/duration of sessions and improving blood purification [20–22].

Some studies have shown that increasing the weekly frequency of dialysis sessions is the best approach to reproduce the kidney physiological functional role, compared to three times/week HD [5, 15, 18, 23]. For instance, Daily HemoDialysis (DHD) consists of more sessions per week (≥5), delivered either as shorter daily sessions (1.5–3 h) [4, 8, 14, 15, 24] or as longer nocturnal sessions (≥5 h) [4, 8, 14, 15, 23, 24]. Some studies on DHD showed that the increased weekly frequency of dialysis has positive effects on the control of blood pressure [4, 8, 14, 19, 20, 23, 24], uremia [6, 10, 15, 19] and ventricular hypertrophy [8, 14, 23–26] in patients with ESRD.

Several studies have also analyzed DHD effects on survival [22, 23, 27–30]. Although most of them found that survival was improved in patients undergoing DHD [22, 28–30], the study by Suri et al. reported that the mortality rate was significantly higher among patients treated with DHD than in those who received conventional HD in in-center HD services [27]. These authors used data from the International Quotidian Dialysis Registry (IQDR) that includes data from many different registries, such as the French Renal Epidemiological and Information Network (REIN) registry. As two-thirds of the data of this study were extracted from the REIN registry, the patients were mainly French, differently from other studies where data, and thus patients, were mainly from the American USRDS database [22, 28, 30]. Therefore, Suri and colleagues hypothesized that the French patients on DHD were older and with more comorbid conditions than patients on conventional HD [27].

In France, patients undergoing DHD represented 1 % of all prevalent cases in 2012 with regional disparities ranging from 0 to 6 % [31]. However, and as mentioned by Suri and colleagues, there are few information on DHD practice and on the demographic and clinical characteristics of the patients undergoing DHD in France. Moreover, the reasons leading to choose DHD among the different RRT options are not described. Nevertheless, literature data suggest that in France, DHD is proposed both to young/active and old/frail patients.

Therefore, to better understand DHD practice in France and in a view of future establishment of recommendations for DHD by the nephrologist community, the aim of this study was to determine the demographic and clinical characteristics of patients on DHD. In addition, the treatment trajectories before starting such a treatment and also the different DHD protocols currently proposed in France will be studied.

## Methods

### Study population

The French REIN registry includes all patients with ESRD undergoing RRT, dialysis or transplantation and living in France. The registry was established in 2002 and since 2011 it covers the entire French territory. The registry organization has been described in details elsewhere [32].

This study was a retrospective analysis of data collected in the REIN registry. For this work, we included all patients aged 18 years and over who started DHD in the French regions included in the REIN registry between January 1, 2003 and December 31, 2012. We excluded patients who initiated RRT in a French region before the introduction of the registry (left truncated data), those who received pre-emptive renal transplantation and those undergoing DHD seven days per week. Each patient was then followed until death or the study end point (December 31, 2013).

### Data collection

#### Patients’ and HD characteristics

To describe the patients’ characteristics at the first DHD, two types of variables were collected: i) demographic data: sex, age; ii) clinical data: smoking status (current/former smoker and never smoked); Body Mass Index (BMI); hemoglobin and albumin levels; comorbidities and disabilities, such as cardiovascular diseases (coronary artery disease, peripheral vascular disease, congestive heart failure, arrhythmia, aneurism and cerebrovascular disease), active malignancy, cirrhosis, respiratory disease, physical or psychiatric disabilities and walking disability (walks without help, needs partial assistance for transfers, totally dependent for transfers). All clinical data were collected at RRT initiation, then annually. Quantitative variables were grouped in clinically relevant classes.

DHD characteristics were described as: i) treatment modality: conventional (hemodialysis) HD and DHD, convective (hemodiafiltration, hemofiltration, biofiltration) HD and DHD, peritoneal dialysis (PD); ii) treatment implementation autonomy: autonomous (home and HD in satellite units and non-assisted PD), and non-autonomous dialysis (nurse-assisted PD, in-center HD; iii) number of weekly sessions and duration of each session; iv) vascular access (catheter, arterio-venous fistula, bypass); v) DHD environment: in-center unit (hospital-based or medically supervised unit); satellite unit (self-care or nurse-assisted) and at home; vi) emergency start vs planned first dialysis session. Treatment modalities were collected at RRT initiation and every time the treatment modality was changed.

#### Treatment trajectories

To describe the patients’ treatment trajectories before DHD initiation, several data included in the REIN registry were used: i) date of the first RRT and treatment modalities; ii) dialysis modality changes before DHD; iii) date of DHD initiation. Additionally, the clinical outcome at the end of the follow-up period (31/12/2013) was included: death; DHD; switch to <5 sessions/week HD; PD; and kidney transplantation.

### Statistical analysis

The patients’ characteristics at DHD initiation were expressed as frequencies and percentages for categorical variables, and as median and interquartile values (IQR) for continuous variables. Demographic and clinical features as well as DHD modalities were described by subgroups, based on the population’s median age, and according to the treatment trajectories before DHD initiation (patients directly on DHD and patients who had other RRT before switching to DHD). The patients’ treatment trajectories after DHD initiation were also described. The characteristics of different subgroups were compared using a Khi-square test.

Patient survival and the access to renal transplantation were assessed from dialysis initiation until death or December, 31 2013. Kaplan Meier survival curves have been established. The Cox regression was applied to evaluate the association between patients’ characteristics and death in the total population and the access to renal transplantation for patients less than 80 years. All variables associated with outcomes in unadjusted model (*p* < 0.2) were included in an adjusted one. All variables presenting a *p*-value < 0.05 in final adjusted model were considered as statistically significant.

Statistical analyses were performed with the Stata 11.1 software (College Station, TX).

## Results

Between 2003 and 2012, 753 patients aged 18 years and over started DHD in the 26 French regions contributing to the REIN registry.

### Demographic and clinical features

Table 1(a) and (b) summarize the demographic and clinical characteristics of patients on DHD. Their mean age at DHD initiation was 62 ± 17.7 years and 63.5 % of them were male. Many patients had one or more comorbidities at DHD start: diabetes (38.6 % of all patients), respiratory disease (15.4 %), active malignancy (13.4 %), one or more cardiovascular diseases (60 %), or physical or psychiatric disabilities (19.5 %).

**Table 1:** Demographical, biological (a) and clinical characteristics (b) at DHD initiation: whole population of patients with ESRD and young and old group based on the median age (<64 vs ≥ 64 years)

(a)	Whole population	<64 years	≥64 years	
	(*n* = 753)	(*n* = 376)	(*n* = 377)	
	n (%)	n (%)	n (%)	*p*
Gender				0.42
Male	478 (63.5)	244 (35)	234 (62)	
Female	275 (36.5)	132 (65)	143 (38)	
BMI (kg/m^2^)^a^				0.40
< 18.5	50 (6.6)	31 (8.2)	19 (5)	
18.5–23	158 (21)	82 (21.8)	76 (20.2)	
23–25	105 (13.9)	53 (14.2)	52 (13.8)	
≥ 25	330 (43.8)	158 (42)	172 (45.6)	
Missing	110 (14.6)	52 (13.8)	58 (15.4)	
Albumin (g/dl)				<0.001
< 30	154 (20.5)	54 (14.4)	100 (26.5)	
≥ 30	490 (65.1)	265 (70.5)	225 (59.7)	
Missing	109 (14.5)	57 (15.2)	52 (13.8)	
Hemoglobin (g/dl)				0.26
< 10	237 (31.5)	118 (31.4)	119 (31.6)	
10–12	295 (39.2)	137 (36.4)	158 (41.9)	
> 12	172 (22.8)	92 (24.5)	80 (21.2)	
Missing	49 (6.5)	29 (7.7)	20 (5.3)	
Smoking status				0.02
Current/former smokers	305 (40.5)	166 (44)	139 (37)	
Never smoker	366 (48.6)	180 (48)	186 (49.3)	
Missing	82 (10.9)	30 (8)	52 (13.7)	
(b)	Whole population	<64 years	≥64 years	
	(*n* = 753)	(*n* = 376)	(*n* = 377)	
	n (%)	n (%)	n (%)	p
Diabetes				<0.001
Yes	291 (38.7)	109 (29)	182 (48.3)	
No	455 (60.4)	265 (70.5)	190 (50.4)	
Missing	7 (0.9)	2 (0.5)	5 (1.3)	
Active malignancy^b^				0.002
Yes	101 (13.4)	36 (9.6)	65 (17.2)	
No	638 (84.7)	336 (89.4)	302 (80)	
Missing	14 (1.9)	1 (1)	10 (2.8)	
Respiratory Disease				0.01
Yes	116 (15.4)	45 (12)	71 (18.8)	
No	620 (82.3)	325 (86.4)	295 (78.2)	
Missing	17 (2.3)	6 (1.6)	11 (3)	
Hepatic disease				0.10
Yes	24 (3.2)	14 (3.7)	10 (2.6)	
No	713 (94.7)	358 (95.3)	355 (94.2)	
Missing	16 (2.1)	4 (1)	12 (3.2)	
Cardiovascular Disease^c^				<0.001
0	297 (39.4)	222 (59)	75 (20)	
1	160 (21.2)	69 (18.4)	91 (24)	
2	125 (16.6)	36 (9.6)	89 (23.6)	
> 2	171 (22.7)	49 (13)	122 (32.4)	
Physical and/or Psychiatric Disabilities				0.10
Yes	147 (19.5)	73 (19.4)	74 (19.5)	
No	567 (75.3)	290 (77)	277 (73.5)	
Missing	39 (5.2)	13 (3.5)	26 (7)	
Walking disability				<0.001
Walks without help	502 (66.7)	299 (79.6)	203 (53.8)	
Totally dependent for transfer	71 (9.4)	23 (6)	48 (12.7)	
Needs assistance for transfer	92 (12.2)	19 (5)	73 (19.5)	
Missing	88 (11.7)	35 (9.4)	53 (14)	
Treatment modality^d^				<0.001
sDHD	496 (66)	278 (74)	218 (58)	
dDHD	257 (34)	98 (26)	159 (42)	

^a^ *BMI* Body Mass Index

^b^Active malignancy: solid tumors or hematological malignancies; ^c^Cardiovascular Disease: myocardial infarction, arrhythmias, coronary insufficiency, heart failure, arteritis of the lower limbs, cerebrovascular accident; ^d^sDHD: patients who switched to DHD after other dialysis modalities; dDHD: patients who started directly with DHD

Then, patients were divided in two groups based on their median age at DHD initiation (64 years; Additional file 1: Figure S1): young (<64 years; *n* = 376) and old (≥64 years; *n* = 377) group. The patients’ mean age in the young group was 47.2 ± 12 years and in the old group was 76.6 ± 7 years. As expected, more patients in the old group had comorbidities than in the young group (Table 1(b)): diabetes (48.3 % *vs* 29 %, *p* < 0.001), active malignancy (17.2 % *vs* 9.6 %, *p* = 0.002), one or more cardiovascular disease (80 % *vs* 40 %, *p* < 0.001) and had more frequently severe walking disabilities (12.7 % *vs* 6 %, *p* < 0.001).

### Patients’ treatment trajectories before starting DHD

Then, patients were divided in two groups based on their pre-DHD trajectory: patients who started RRT directly with DHD (dDHD; *n* = 257) and patients who started with other RRT before switching to DHD (**s**DHD; *n* = 496). The percentage of patients aged ≥64 years was higher in the dDHD than in the sDHD group (62 % *vs* 44 %) as well as the proportion of patients with comorbidities or in bad clinical conditions (Table 2). Specifically, albumin levels were <30 g/dl in 33.5 % of patients in the dDHD group (*vs* 13.7 % in the sDHD group) and hemoglobin levels <10 g/dl were detected in 47.9 % of patients in the dDHD group (*vs* 23 % in the sDHD group). Moreover, more patients in the dDHD group had an active malignancy (18.3 % *vs* 10.9 % in the sDHD group, *p* < 0.001) or serious walking disabilities (16.3 % *vs* 5.8 % in the sDHD group, *p* < 0.001).

**Table 2:** Demographic, biological characteristics (a) and comorbidities (b) of patients based on their treatment trajectory (sDHD and dDHD) at DHD initiation

(a)	sDHD patients	dDHD patients^d^	
	(*n* = 496)	(*n* = 257)	
	n (%)	n (%)	*p*
Gender			0.002
Male	330 (66.5)	148 (57.6)	
Female	166 (33.5)	109 (42.4)	
Age (years)			<0.001
< 64	278 (56)	98 (38)	
≥ 64	218 (44)	159 (62)	
BMI (kg/m^2^)^a^			<0.001
< 18.5	27 (5.4)	23 (8.9)	
18.5–23	106 (21.4)	52 (20.2)	
23–25	70 (14.1)	35 (13.6)	
≥ 25	249 (50.2)	81 (31.5)	
Missing	44 (8.9)	66 (25.7)	
Albumin (g/dl)			<0.001
< 30	68 (13.7)	86 (33.5)	
≥ 30	374 (75.4)	116 (45.1)	
Missing	54 (10.9)	55 (21.4)	
Hemoglobin (g/dl)			<0.001
< 10	114 (23)	123 (47.9)	
10–12	226 (45.6)	69 (26.8)	
> 12	135 (27.2)	37 (14.4)	
Missing	21 (4.2)	28 (10.9)	
Smoking status			0.003
Current/former smokers	220 (44.4)	85 (33.1)	
Never smoker	232 (46.8)	134 (52.1)	
Missing	44 (8.9)	38 (14.8)	
(b)	sDHD patients	dDHD patients^d^	
	(*n* = 496)	(*n* = 257)	
	n (%)	n (%)	*p*
Diabetes			0.001
Yes	194 (39)	97 (37.7)	
No	302 (61)	153 (59.5)	
Missing	0 (0)	7 (2.7)	
Active malignancy^b^			<0.001
Yes	54 (10.9)	47 (18.3)	
No	439 (88.5)	199 (77.4)	
Missing	3 (0.6)	11 (4.3)	
Respiratory Disease			<0.001
Yes	81 (16.3)	35 (13.6)	
No	412 (83.1)	208 (80.9)	
Missing	3 (0.6)	14 (5.4)	
Hepatic disease			<0.001
Yes	17 (3.4)	7 (2.7)	
No	476 (96)	237 (92.2)	
Missing	3 (0.6)	13 (5.1)	
Cardiovascular Disease^c^			0.25
0	206 (41.5)	91 (35.4)	
1	96 (19.4)	64 (24.9)	
2	82 (16.5)	43 (16.7)	
> 2	112 (22.6)	59 (23)	
Physical and/or Psychiatric Disabilities			0.001
Yes	89 (17.9)	58 (22.6)	
No	391 (78.8)	176 (68.5)	
Missing	16 (3.2)	23 (8.9)	
Walking disability			<0.001
Walks without help	373 (75.2)	129 (50.2)	
Totally dependent for transfer	29 (5.8)	42 (16.3)	
Needs assistance for transfer	55 (11.1)	37 (14.4)	
Missing	39 (7.9)	49 (19.1)	

^*a*^ *BMI* Body Mass Index

^b^Active malignancy: solid tumors or hematological malignancies; ^c^Cardiovascular Disease: myocardial infarction, arrhythmias, coronary insufficiency, heart failure, arteritis of the lower limbs, cerebrovascular accident; ^d^sDHD: patients who switched to DHD after other dialysis modalities; dDHD: patients who started directly with DHD

Patient in the sDHD group switched to DHD after a median RRT duration of 2 years (IQR: 1–3.8). During this period, 27 (5.4 %) changed at least once their dialysis modality. Specifically, 19 patients switched from PD to HD and eight patients from HD to PD. Only three patients changed twice their treatment modality before switching to DHD. Conventional HD was the pre-DHD treatment of choice among patients in the sDHD group (82.7 %; *n* = 410), followed by PD (9.9 %; *n* = 49) and convective HD (7.5 %; *n* = 37). Moreover, non-autonomous dialysis, mainly in-center dialysis requiring partial or total nurse assistance, was frequent in the sDHD group (79 % of patients).

### DHD regimens

At DHD initiation, 602 patients (80 %) had conventional DHD (Table 3) with five sessions/week (24.4 %) or six sessions/week (55.6 %). The others (20 %) had five sessions/week (5.6 %) or six sessions/week (14.4 %) of convective DHD. The session’s median duration was 3 h (IQR: 2–3). Only seven patients had sessions longer than five hours.

**Table 3:** DHD features

	Whole population	sDHD patients	dDHD patients^b^	
	(*n* = 753)	(*n* = 496)	(*n* = 257)	
	n (%)	n (%)	n (%)	*p*
Autonomy				0.001
Autonomous	134 (18.8)	123 (24.5)	11 (4.3)	
Non-autonomous	619 (82.2)	373 (75.2)	246 (95.7)	
DHD modalities^a^				0.01
coDHD	602 (80)	383 (77.2)	219 (85.2)	
cvDHD	151 (20)	113 (22.8)	38 (14.8)	
Vascular access				<0.001
Catheter	192 (25.5)	80 (16.1)	112 (43.6)	
Arterio-venous fistula	378 (50.2)	326 (65.7)	52 (20.2)	
Bypass	9 (1.2)	9 (1.8)	0 (0.0)	
Other	26 (3.5)	6 (1.2)	20 (7.8)	
DHD environment				<0.001
At home	43 (5.8)	40 (8.1)	3 (1.2)	
In-center	547 (72.6)	309 (62.3)	238 (92.6)	
Satellite unit	163 (21.6)	147 (29.6)	16 (6.2)	

^a^coDHD: conventional DHD, *cvDHD* convective DHD, ^b^sDHD: patients who switched to DHD after other dialysis modalities; dDHD: patients who started directly with DHD

Among the patients in the dDHD group, 146 (57 %) initiated DHD in emergency conditions compared to 140 (28 %) in the sDHD group. Patients in the dDHD group had dialysis mainly in in-center HD services (92.6 %), while patients in the sDHD group were dialyzed more often in satellite units (29.6 %) or at home (8.1 %).

### Clinical outcome at the end of the study

**Fig. 1:**
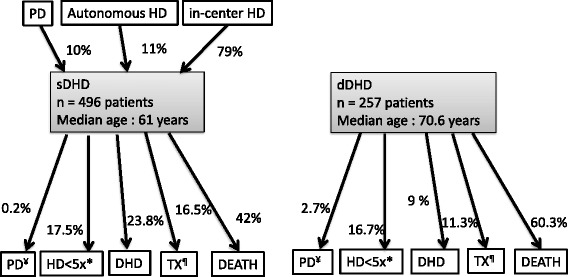
Patients’ treatment trajectories and outcome, by subgroups (sDHD: patients who switched to DHD after other dialysis modalities; dDHD: patients who started directly with DHD). ^¥^PD: Peritoneal Dialysis; *HD < 5x: Hemodialysis <5sessions/week; ^¶^TX: transplantation

Patients underwent DHD for a median duration of 258 days (IQR: 52–487). At 12/31/2013, 363 patients were dead (48.2 %), 141 patients (18.8 %) were still on DHD, 14.7 % underwent kidney transplantation, 17.3 % switched to HD (<5 sessions per week) and 1.1 % to PD (Fig. 1).

#### Survival

The percentage of patients who died was higher in the old group (69 %) than in the young group (27.4 %) and dDHD patients died in higher proportion than sDHD ones (60.3 % *vs* 42 %). The risk of dying at 2 years after dialysis initiation was approximately of 12 % in sDHD patients and of 50 % in dDHD ones (Additional file 1: Figure S2 (a)). The mortality rate was high among old dDHD patients (Additional file 1: Figure S2 (b)). As expected, all major comorbidities were associated with an increased risk of death. In addition, there was a significant increased risk of death for dDHD (HR = 2.91 95 % CI: 2.36–3.6) and this association remained significant in the adjusted model (Additional file 1: Table S1).

#### Renal transplantation

Patients older than 80 years (*n* = 102; 13.5 %) were excluded from the analysis of the access to renal transplantation. During the follow-up, 37/205 dDHD (18 %) and 102/446 sDHD (23 %) patients were transplanted. dDHD patients had a higher access to renal transplantation (Additional file 1: Figure S3 (a)) in the unadjusted as in the adjusted Cox model (HR = 2.03 95 % CI: 1.38–3.0; Additional file 1: Table S2), and especially patients <64 years (Additional file 1: Figure S3 (b)).

## Discussion

This first study on all French patients who started DHD between 2003 and 2012 shows the heterogeneity of the demographic/clinical features and treatment trajectories of this population. Our results indicate that in France, DHD is proposed to old and frail patients with comorbidities who died in high proportion and also to young patients with ESRD who are waiting for kidney transplantation. Moreover, based on their treatment trajectory, patients could be divided in two groups: patients who started directly with DHD (older and with many comorbidities) and patients who started with other RRT before switching to DHD (younger, healthier and waiting for renal transplantation). Finally, our analysis shows that DHD modalities in France are various and depend on the individual patient’s characteristics and pre-DHD treatment trajectories. The heterogeneity of the profile of French patients undergoing DHD could partially explain the contradicting results on mortality in DHD reported by Suri and colleagues [27] compared to other studies [22, 28–30].

The present work is the first study to include patients with ESRD on DHD from all France regions over a long period. To characterize this population, all major comorbidities and disabilities were taken into account. Additionally, the REIN registry includes also data on the treatment modalities as well as on the pre- and post-DHD treatment switches. Therefore, and differently from previous studies that only described the first RRT modality and/or the length of the pre-DHD period [16, 18, 22, 28, 33], we could analyze and describe all the RRT modality changes before switching to DHD.

We extracted from the REIN registry information on several comorbidities in order to precisely describe the clinical status of patients on DHD. We found that diabetes (38.6 % of all patients), cardiovascular diseases (60 %) and other comorbidities (respiratory disease: 15.4 %, active malignancy: 13.4 %, walking disabilities: 21.6 %) are frequent among French patients undergoing DHD. Previous studies focused mainly on cardiac functions [8, 16, 25, 26] and less frequently on other comorbidities [22, 27, 28, 30, 34], such as diabetes. Indeed, cardiovascular complications are the leading cause of mortality in patients with ESRD and the usual reason for proposing DHD. Moreover, previous studies reported lower percentages of patients on DHD with comorbidities: between 19 and 32 % for diabetes [27, 28], and between 12 and 31 % for cardiovascular diseases [27, 34]. In addition, our study indicates that French patients on DHD are older (mean age: 62 years) than patients in previous studies that assessed the effect of DHD on survival (mean age: from 35.6 to 55.8 years) [27, 35]. These findings may in part explain the higher mortality rate among patients undergoing DHD compared with conventional HD (HR = 1.3; *p* = 0.034) reported by Suri and colleagues [27] and in disagreement with previous reports [22, 28–30, 33]. Indeed, our study highlights that a large proportion of French patients on DHD are older and suffer from comorbidities, differently from the patients included in other works. Inversely, they accessed more to renal transplantation. Comparison with a similar population of patients undergoing DHD is now essential to definitively conclude on the survival benefit associated with DHD.

Patients in the dDHD group were more often dialyzed via a catheter (43.6 %) than those in the sDHD group (20.4 %) and started more frequently the treatment in emergency conditions (57 % vs 28 %). Starting DHD in an emergency situation often requires dialysis via a catheter. This could explain why arterio-venous fistulae were less common in our population (35.6 %) compared with previous studies where vascular access by arterio-venous fistula was used in 63 % [18; 27] to 89 % [35] of patients. Moreover, 20 % of all our patients had albumin levels <30 g/dl and 31 % hemoglobin levels <10 g/dl, suggesting that our population is predisposed to malnutrition [21, 23] and anemia [5, 21, 24]. The finding that 48 and 33.5 % of patients in the dDHD group had low hemoglobin and albumin levels, respectively, indicates, as reported in previous studies [9, 18, 21, 24, 36], that patients started DHD as an emergency because of their poor medical status which did not allow proposing thrice-weekly HD with long sessions. Our results showed that patients who started directly with DHD presented a higher risk of mortality (HR = 2.44; 95 % CI: 1.91–3.11), but paradoxically they had a better access to renal transplantation (HR = 2.03; 95 % CI: 1.38–3.0). These results suggested that profiles of DHD patients in France are heterogeneous: dDHD is the first RRT of old and frail patients as well as younger ones in better medical conditions.

The association between DHD and biological parameters was impossible to measure in our study. Nevertheless, it has been reported that DHD improves the control of hypertension [26]. DHD is also associated with improved fluid and phosphorus management and inflammatory factors [37]. No negative impacts of DHD on residual renal function have been observed [38]. Arterio-venous fistula is the favorite vascular access for DHD [18], and no statistically significant differences in vascular access dysfunction or permanent failures among DHD patients compared to conventional HD ones were reported [39]. Finally, improvements of quality of life with DHD have been largely studied [4, 8, 10, 11, 20, 21].

In the literature, the RRT trajectory of patients on DHD has not been well described. In our study, patients in the sDHD group had mainly thrice-weekly HD (81 %) or PD (10 %) before switching to DHD. Only a third of all our patients started directly with DHD. Among the 496 French patients who switched to DHD, 21 % had autonomous dialysis requiring little nurse assistance and therefore had fewer constraints than patients dialyzed in outpatient units. Moreover, younger patients had often a past history of autonomous dialysis before starting DHD. Dialysis at home is associated with a better quality of life and self-rehabilitation [6] and it is often proposed to young patients in good clinical conditions [6, 33, 36]. Younger patients dialyzed at home have fewer comorbidities [28] and more access to renal transplantation than older patients [22]. These observations could explain the association of DHD with improved patient survival. Indeed, these studies included patients in quite good clinical conditions who expected to maintain their social and professional lives, while being dialyzed every day at home [4, 22, 28].

In France, the most frequently prescribed DHD regimen is HD (six 3 h-sessions per week). This protocol has been extensively assessed and it is considered the best choice [4–6, 24] to limit the large fluctuations of body fluid volumes caused by thrice-weekly HD [4, 24]. Our results show that long nocturnal hemodialysis is rarely used in France. Indeed, only 0.9 % of all patients underwent five to six night sessions of more than five hours/each per week. Nocturnal hemodialysis at home is much developed in Canada and Australia to overcome the geographical isolation of dialyzed patients [40]. Such patients are remote-monitored during the night and alerted if a problem that needs their intervention arises [23, 36]. Home dialysis requires specific equipment and organization. On the other hand, in France, there are many dialysis facilities with satellite dialysis units [41] to meet the patients’ demands in all regions and to avoid patients’ isolation as observed in Canada and Australia.

The provision of care for patients in dialysis has an important economic impact on the Health Insurance in France as in the whole world. Costs associated with medical supplies are more important for in-center DHD [4, 42, 43] than out-center or home HD due to staff and transport costs (reimbursed in France) [24, 44]. However, the development of low-flux machines at home may reduce the costs of DHD.

The main limitation of our study is the lack in the REIN registry of the medical reasons to explain the nephrologist’s decision to start or to switch a patient to DHD, differently from the USRDS registry where such reasons are described. Moreover, data from REIN didn’t allow us to evaluate the quality of life of patients included in the study.

## Conclusions

Our analysis of the demographic/clinical features and treatment trajectories of 753 French patients on DHD shows that in France, DHD is administered to aged patients with multiple comorbidities and high mortality and also to relatively younger patients on the waiting list for renal transplantation. The heterogeneity of the patients’ profiles suggests that DHD indications in France are various. Moreover, the data on the patients’ demographic and clinical characteristics and on their initial RRT trajectories could be further used to analyze the association between DHD and patient survival by taking into account their comorbidities and access to renal transplantation.

## Abbreviations

dDHD, starting directly with DHD; DHD, daily hemodialysis; ESRD, end stage renal disease; HD, hemodialysis; HR, hazard ratio; IQR, interquartile range; PD, peritoneal disease; RRT, renal replacement therapy; sDHD, starting with other dialysis before switching to DHD

## Additional file

Additional file 1: Supplementary material. **Figure S1.** Age distribution of patient at DHD initiation. **Figure S2.** (a,b). Survival curves of patients by subgroups (trajectory, median age combined with trajectory). **Table S1.** Factors associated with survival. **Figure S3.** (a,b). Cumulative incidence function for the access to renal transplantation patients by subgroups (trajectory, median age combined with trajectory). **Table S2.** Factors associated with renal transplantation. (DOCX 100 kb)
