# Supplementary material for: French patients on daily hemodialysis: clinical characteristics and treatment trajectories
Source: BMC Nephrol. 2016 Jul 29;17:107. doi: 10.1186/s12882-016-0306-7 (PMC4966797; doi:10.1186/s12882-016-0306-7)
Supplement: Supplementary file 1 — Supplementary material. Figure S1. Age distribution of patient at DHD initiation. Figure S2. (a,b). Survival curves of patients by subgroups (trajectory, median age combined with trajectory). Table S1. Factors associated with survival. Figure S3. (a,b). Cumulative incidence function for the access to renal transplantation patients by subgroups (trajectory, median age combined with trajectory). Table S2. Factors associated with renal transplantation. (DOCX 100 kb) [file 12882_2016_306_MOESM1_ESM.docx]

**Additional file 1**


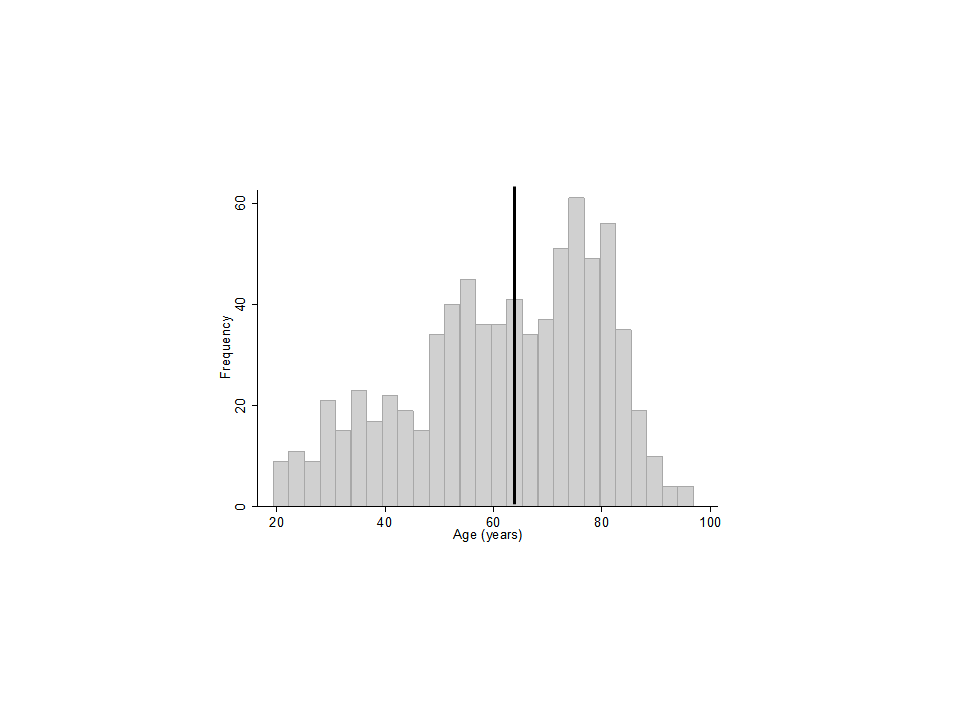


**Figure S1. Histogram of age at DHD initiation, median age: 64 years (vertical solid line)**

**
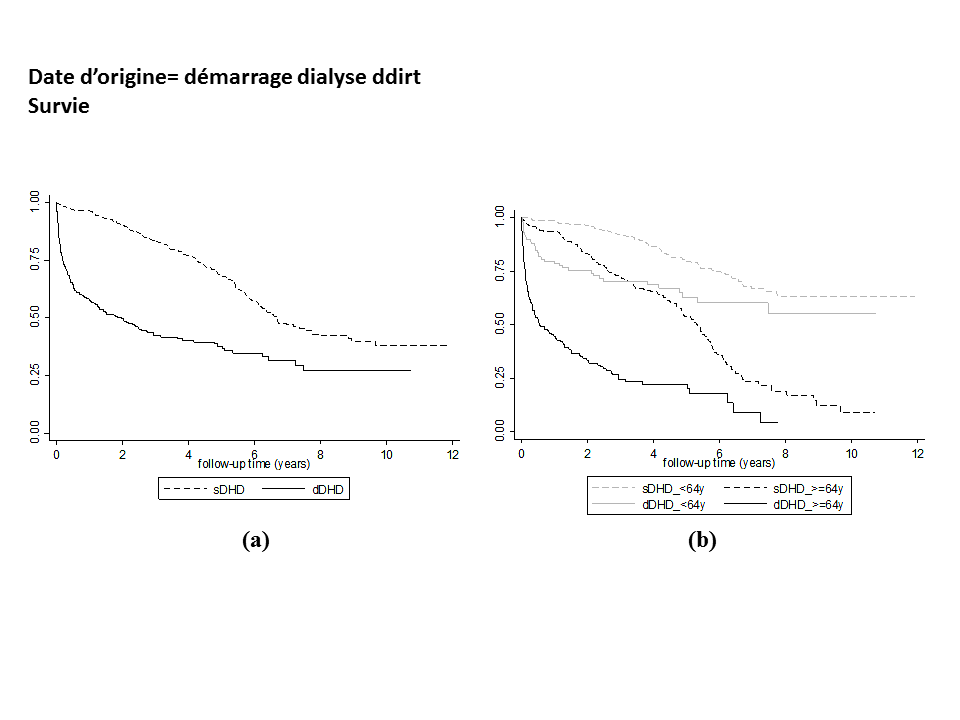
Figure S2. Survival curves according to (a) trajectory subgroups: dDHD (dash line), sDHD (dote line); (b) median age combined with trajectory groups: age<64 years (grey), age ≥64 years (black).**

**Table S1. Factors associated with survival: univariate and multivariate Cox model.**

|  | **Univariate Cox** | | **Multivariate Cox** | |
| --- | --- | --- | --- | --- |
|  | **HR (95%CI )** | **p** | **HR (95%CI )** | **p** |
| **Sex** (vs men) | 1.17 (0.94-1.44) | 0.153 | 1.05 (0.84-1.33) | 0.649 |
| **Age** (vs 18-40 years) |  |  |  |  |
| 40-60 | 6.50 (3.06-13.8) | <0.0001 | 5.55 (2.63-11.7) | <0.0001 |
| 60-70 | 11.06 (5.1-23.9) | <0.0001 | 9.8 (4.56-21.03) | <0.0001 |
| ≥70 | 18.5 (8.8-38.8) | <0.0001 | 16.6 (7.9-34.6) | <0.0001 |
| **Albumin** (vs ≥30 g/dl) |  |  |  |  |
| <30 | 2.63 (2.06-3.35) | <0.0001 | 1.43 (1.09-1.9) | 0.01 |
| Missing | 1.35 (0.99-1.83) | 0.053 | 1.41 (0.97-2.07) | 0.074 |
| **Hemoglobin** (vs 10-12 g/dl) |  |  |  |  |
| <10 | 1.69 (1.32-2.15) | <0.0001 | 1.46 (1.12-1.90) | 0.005 |
| >12 | 0.80 (0.61-1.07) | 0.129 | 0.86 (0.64-1.15) | 0.302 |
| Missing | 0.94 (0.59-1.51) | 0.807 | 0.79 (0.44-1.43) | 0.439 |
| **BMI** (vs 23-25 kg/m²) |  |  |  |  |
| <18.5 | 1.54 (0.97-2.45) | 0.068 | - | - |
| (18.5-23) | 1.07 (0.74-1.55) | 0.709 | - | - |
| ≥25 | 1.03 (0.74-1.43) | 0.88 | - | - |
| Missing | 1.83 (1.25-2.68) | 0.002 | - | - |
| **Smoking status** (vs never-smoker) |  |  |  |  |
| Current/former smoker | 0.94 (0.76-1.18) | 0.613 | - | - |
| Missing | 1.65 (1.20-2.26) | 0.002 | - | - |
| **Diabetes** (vs no) |  |  |  |  |
| Yes | 1.65 (1.34-2.04) | <0.0001 | - | - |
| Missing | 10.4 (4.57-23.75) | <0.0001 | - | - |
| **Active malignancy** (vs no) |  |  |  |  |
| Yes | 1.37 (1.03-1.81) | 0.029 | - | - |
| Missing | 3.25 (1.73-11.5) | <0.0001 | - | - |
| **Respiratory disease** (vs no) |  |  |  |  |
| Yes | 1.69 (1.31-2.17) | <0.0001 | 1.36 (1.04-1.77) | 0.023 |
| Missing | 4.90 (2.73-8.81) | <0.0001 | 0.55 (0.22-1.38) | 0.204 |
| **Hepatic disease** (vs no) |  |  |  |  |
| Yes | 2.23 (1.37-3.63) | 0.001 | 2.62 (1.58-4.36) | <0.0001 |
| Missing | 6.57 (3.74-11.5) | <0.0001 | 4.50 (1.88-10.7) | 0.001 |
| **Cardiovascular diseases** (vs 0) |  |  |  |  |
| 1 | 2.14 (1.58-2.89) | <0.0001 | 1.27 (0.92-1.74) | 0.15 |
| 2 | 2.44 (1.78-3.34) | <0.0001 | 1.30 (0.92-1.83) | 0.136 |
| >2 | 3.28 (2.47-4.34) | <0.0001 | 1.66 (1.20-2.28) | 0.002 |
| **Walking disabilitites** (vs no) |  |  |  |  |
| Totally dependent | 3.55 (2.60-4.85) | <0.0001 | 1.87 (1.33-2.62) | <0.0001 |
| Needing assicstance | 3.10 (2.35-4.09) | <0.0001 | 1.61 (1.20-2.17) | 0.002 |
| Missing | 2.98 (2.23-3.99) | <0.0001 | 3.04 (2.20-4.20) | <0.0001 |
| **Physical/Psychiatric disabilities** (vs no) |  |  |  |  |
| Yes | 1.27 (0.98-1.63) | 0.068 | - | - |
| Missing | 2.60 (1.78-3.80) | <0.0001 | - | - |
| **DHD initiation** (vs sDHD) |  |  |  |  |
| dDHD | 2.91 (2.36-3.60) | <0.0001 | 2.44 (1.91-3.11) | <0.0001 |

**
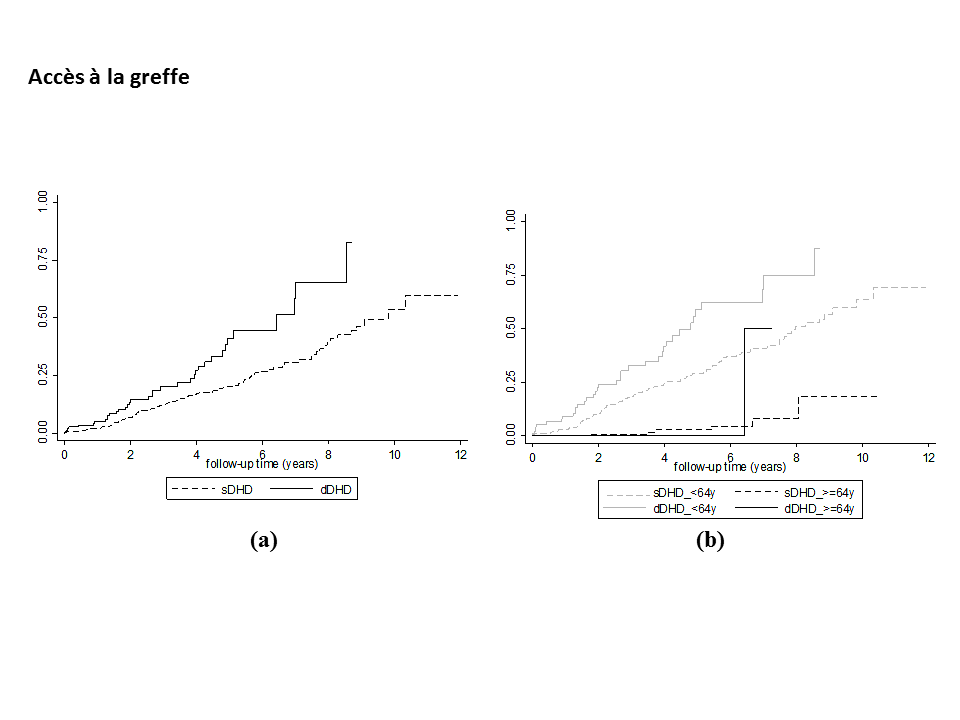
**

**Figure S3. Access to renal transplantation according to (a) trajectory subgroups: dDHD (dash line), sDHD (dote line); (b) median age combined with trajectory groups: age<64 years (grey), age ≥64 years (black).**

**Table S2. Factors associated with renal transplantation at the endpoint in patients <81 years (n= 669): univariate and multivariate Cox model.**

|  | **Univariate Cox** | | **Multivariate Cox** | |
| --- | --- | --- | --- | --- |
|  | **HR (95%CI )** | **p** | **HR (95%CI )** | **p** |
| **Sex** (vs men) | 0.87 (0.61-1.24) | 0.44 | 0.73 (0.51-1.06) | 0.095 |
| **Age** (vs 18-40 years) |  |  |  |  |
| 40-60 | 0.46 (0.32-0.64) | <0.0001 | 0.42 (0.30-0.60) | <0.0001 |
| 60-70 | 0.13 (0.07-0.26) | <0.0001 | 0.14 (0.07-0.28) | <0.0001 |
| 70-80 | 0.01 (0.001-0.07) | <0.0001 | 0.01 (0.01-0.07) | <0.0001 |
| **Albumin** (vs ≥30 g/dl) |  |  |  |  |
| <30 | 1.27 (0.75-2.14) | 0.37 | - | - |
| Missing | 1.50 (0.97-2.31) | 0.068 | - | - |
| **Hemoglobin** (vs 10-12 g/dl) |  |  |  |  |
| <10 | 1.42 (0.92-2.18) | 0.113 | - | - |
| >12 | 1.20 (0.79-1.81) | 0.39 | - | - |
| Missing | 1.52 (0.81-2.85) | 0.189 | - | - |
| **BMI** (vs 23-25 kg/m²) |  |  |  |  |
| <18.5 | 0.85 (0.38-1.88) | 0.681 | - | - |
| (18.5-23) | 0.78 (0.46-1.33) | 0.36 | - | - |
| ≥25 | 0.70 (0.44-1.13) | 0.146 | - | - |
| Missing | 1.29 (0.72-2.31) | 0.386 | - | - |
| **Smoking status** (vs never-smoker) |  |  |  |  |
| Current/former smoker | 0.87 (0.62-1.24) | 0.449 | - | - |
| Missing | 0.94 (0.50-1.78) | 0.859 | - | - |
| **Diabetes** (vs no) |  |  |  |  |
| Yes | 0.40 (0.26-0.62) | <0.0001 | - | - |
| Missing | - | - | - | - |
| **Hepatic disease** (vs no) |  |  |  |  |
| Yes | 0.64 (0.16-2.60) | 0.536 | - | - |
| Missing | - | - | - | - |
| **Active malignancy** (vs no) |  |  |  |  |
| Yes | 0.61 (0.33-1.13) | 0.119 | - | - |
| Missing | - | - | - | - |
| **Respiratory disease** (vs no) |  |  |  |  |
| Yes | 0.34 (0.16-0.72) | 0.005 | - | - |
| Missing | - | - | - | - |
| **Cardiovascular diseases** (vs 0) |  |  |  |  |
| 1 | 0.52 (0.33-0.80) | 0.003 | - | - |
| 2 | 0.20 (0.1-0.42) | <0.0001 | - | - |
| >2 | 0.18 (0.09-0.38) | <0.0001 | - | - |
| **Walking disabilitites** (vs no) |  |  |  |  |
| Totally dependent | 0.34 (0.11-1.07) | 0.065 | - | - |
| Needing assicstance | 0.27 (0.08-0.84) | 0.024 | - | - |
| Missing | 1.30 (0.76-2.22) | 0.346 | - | - |
| **Physical/Psychiatric disabilities** (vs no) |  |  |  |  |
| Yes | 0.42 (0.24-0.74) | 0.003 | 0.43 (0.24-0.76) | 0.004 |
| Missing | 0.33 (0.08-1.35) | 0.125 | 0.26 (0.06-1.05) | 0.058 |
| **DHD initiation** (vs sDHD) |  |  |  |  |
| dDHD | 2.05 (1.40-3.02) | <0.0001 | 2.03 (1.38-3.0) | <0.0001 |
